# Comprehensive analysis of molecular features, prognostic values, and immune landscape association of m6A-regulated immune-related lncRNAs in smoking-associated lung squamous cell carcinoma

**DOI:** 10.3389/fgene.2022.887477

**Published:** 2022-08-10

**Authors:** Meng Zhang, Jian Zhang, Yang Liu

**Affiliations:** ^1^ School of Medicine, Nankai University, Tianjin, China; ^2^ Department of Thoracic Surgery, The First Medical Centre, Chinese PLA General Hospital, Beijing, China

**Keywords:** M6A, immune lncRNA, prognosis, tumor microenvironment, smoking, squamous cell lung carcinoma

## Abstract

Lung squamous cell carcinoma (LUSC) is the second most common histopathological subtype of lung cancer, and smoking is the leading cause of this type of cancer. However, the critical factors that directly affect the survival rate and sensitivity to immunotherapy of smoking LUSC patients are still unknown. Previous studies have highlighted the role of N6-methyladenosine (m6A) RNA modification, the most common epigenetic modification in eukaryotic species, together with immune-related long non-coding RNAs (lncRNAs) in promoting the development and progression of tumors. Thus, elucidating m6A-modified immune lncRNAs in LUSC patients with smoking history is vital. In this study, we described the expression and mutation features of the 24 m6A-related regulators in the smoking-associated LUSC cohort from The Cancer Genome Atlas (TCGA) database. Then, two distinct subtypes based on the expression levels of the prognostic m6A-regulated immune lncRNAs were defined, and differentially expressed genes (DEGs) between the subtypes were identified. The distributions of clinical characteristics and the tumor microenvironment (TME) between clusters were analyzed. Finally, we established a lncRNA-associated risk model and exhaustively clarified the clinical features, prognosis, immune landscape, and drug sensitivity on the basis of this scoring system. Our findings give insight into potential mechanisms of LUSC tumorigenesis and development and provide new ideas in offering LUSC patients with individual and effective immunotherapies.

## Introduction

Lung cancer, one of the leading causes of cancer death all over the world, also has a higher morbidity than other cancer types, seriously imperiling people’s health and lives nowadays. Many key issues have been identified and considered to be closely related to the tumorigenesis of lung cancer, such as smoking, environmental pollution, occupational exposure, and, especially, some genetic factors (Goldstraw et al., 2011). Lung squamous cell carcinoma (LUSC) is the most common histopathological subtype after lung adenocarcinoma (LUAD), accounting for nearly 30% of patients who are diagnosed with lung cancer (Socinski et al., 2018). It is worth mentioning that smoking is the leading cause of LUSC, and most patients with this kind of cancer have a clear history of smoking (Alexandrov et al., 2016). One research focusing on non-smoking lung cancer in Eastern Asia revealed that smoking-related and non-smoking-related lung cancers were quite different, and they were likely to show different responses to targeted therapies (Chen et al., 2020a). In addition, numerous studies have found that smoking could profoundly influence the tumor microenvironment (TME) and lung microbiome, promoting the progression and metastasis of lung cancer (Desricha rd et al., 2018). To have a better understanding of LUSC, much research and clinical trials were dedicated to revealing the molecular mechanisms of the cancer and putting forward some effective measures to decrease the mortality of the disease both in prevention and treatment aspects, including early screening and some particular therapy methods like immunotherapy (Pan et al., 2021). Although these measures may achieve a certain effect in some degree, the 5-year overall survival rate does not markedly improve, especially for these advanced or poorly differentiated tumors. For these reasons, it is necessary to build an effective and accurate risk model to predict the prognosis of LUSC patients with smoking history.

N6-methyladenosine (m6A) RNA modification is a current hotspot in the cancer research area. Although it was first discovered as early as the 1970s, further research was significantly limited by the detection and research techniques those days, and studies on this kind of epigenetic modification have been stagnant for a long time (Wang et al., 2014). With the development of high-throughput sequencing, colorimetry, and liquid chromatography–mass spectrometry techniques (specific techniques including MeRIP-seq, miCLIP-seq, SCARLET, and LC-MS/MS), RNA methylation attracted people’s attention again, and it has now been shown that more than half of the nucleic acid methylation modifications belong to m6A modification. By interacting with various functional proteins, m6A modification can affect the processing of almost all kinds of RNA molecules in multiple species, such as messenger RNA (mRNA; splicing, subcellular localization, polyadenylation, translation, and degradation), small non-coding RNA, and transporter RNA (tRNA) (Cantara et al., 2011; Liu and Jia, 2014; Alarcon et al., 2015; Zhang and Jia, 2018). In addition, according to previous studies, many biological processes such as tissue development, circadian rhythm regulation, DNA damage response, gender determination, and the development and progression of various diseases, especially of tumors, were closely regulated by this sort of epigenetic modification (Lence et al., 2016; Xiang et al., 2017; Zhao et al., 2017; Kasowitz et al., 2018; Tong et al., 2018). Further studies have illuminated that several enzymes or proteins were directly involved in the m6A RNA modification process, and then, these proteins could be divided into three categories based on their special structural features and functions: 1. methyltransferase (writers): a complex composed of multiple subunits to modify m6A methylation on RNA, including a core complex consisting of METTL3 (catalytic subunit) and METTL14 and several subunits that can increase the activity and specificity of the complex, such as WTAP, RBM15, KIAA1429, ZC3H13, and RBM15B; 2. m6A-binding proteins (readers): playing a biological function by recognizing and binding m6A methylation marks on RNA, including YTHDF family of proteins (YTHDF1-3), YTHDC1, YTHDC2, HNRNPC, HNRNPA2B1, EIF3A, and IGF2BPs; 3. demethylases: only two demethylases, FTO and ALKBH5, have been identified so far, which can remove the m6A methyl group from the modulated RNA, although they may exhibit different demethylation activities in a specific tissue or reaction environment (Jiang et al., 2021).

Long non-coding RNAs (lncRNAs), as one of the most important kinds of non-coding RNAs, are longer than 200 nucleotides in length but are not translated into proteins (Esteller, 2011). Although structurally similar to mRNAs, lncRNAs still have some special and unique features such as low conservation, high abundance, and timing specificity. With the development of high-throughput sequencing technology, it is believed that more lncRNAs will be discovered, and their biological functions in diseases, especially in tumorigenesis, will also be elucidated in a new way. Recent research studies have shown that lncRNAs could also undergo m6A methylation modification ultimately affecting their stability, subcellular localization, and local structure, resulting in the alteration of their biological regulatory functions (Chen et al., 2020b). Moreover, lncRNAs were also reported to be involved in the processes of immune response in multiple cancers (Hu et al., 2019). Thus, whether immune-related lncRNAs could be modulated by m6A modification and promote tumor progression in LUSC needs further studies.

TME refers to the inner environment in which tumor cells arise and live. A variety of cells including tumor cells, fibroblasts, immune and inflammatory cells, along with the intercellular substance, microvessels, and immune-associated biological molecules infiltrating in this region, together make up the TME (Liu et al., 2021). In recent years, increasing research has concentrated on the impact of the dynamic changes and related factors of the TME on tumorigenesis and progression of tumors, and multiple mechanisms underlying the TME-promoting tumor development have been exhaustively clarified (Ringquist et al., 2021). As the TME can profoundly influence the progression and strategies of immune therapies for cancer patients, several algorithms based on RNA-seq data to evaluate the composition of immune-related cells in the TME have been developed, including TIMER, CIBERSORT, EPIC, MCP-counter, and ESTIMATE (Xu et al., 2021).

As presented earlier, m6A RNA modification, immune-related lncRNA, and dynamic changes in TME components occupy important positions in the development and progression of tumors. Whether the immune-related lncRNAs regulated by m6A regulators can significantly affect the prognosis of smoking-associated LUSC patients in a coordinated manner through the TME mechanisms deserves further studies, although several comprehensive and systematic studies have demonstrated these complicated relationships in LUAD patients (Zhang et al., 2021; Zheng et al., 2021; Zhou and Gao, 2021). Despite much research existing in the field of LUAD, the study of LUSC in this area has obviously not received enough attention. Although surgery for early LUSC can achieve good therapeutic results, advanced patients do not have many appropriate treatment options on the grounds that this kind of tumor is particularly resistant to radiochemotherapy. Owing to the reasons mentioned earlier, immunotherapy combined with chemotherapy has become the first-line treatment for advanced LUSC patients. Thus, exploring the role of m6A RNA methylation in the immune response of LUSC patients will help develop better immunotherapy strategies.

In this study, we first described the expression profiles and mutation characteristics of the 24 m6A-related proteins in the smoking-associated LUSC cohort from The Cancer Genome Atlas (TCGA) database. Second, lncRNAs that were closely co-expressed with immune genes and m6A regulators were identified. Then, these LUSC patients were classified into different molecular subtypes based on the expression levels of the selected lncRNAs. Gene subtypes were then set up according to the differentially expressed genes (DEGs) of the previously established molecular subtypes. Finally, we established a lncRNA-associated signature as a prognostic model and exhaustively clarified the immune landscape on the basis of this scoring system, aiming to provide insight into the potential immune mechanisms of LUSC tumorigenesis and predicting the prognosis and response of immunotherapy for smoking-associated LUSC patients.

## Materials and methods

### Patients and datasets

The LUSC patients with definite smoking history, including reformed and current smokers in TCGA database, were enrolled in this study. All patients’ information, including RNA-seq data (exhibited as fragments per kilobase million, FPKM), corresponding clinical information (age, gender, TNM (tumor node metastasis classification) stage, T (tumor), N (node), M (metastasis), smoking history, overall survival, and survival state), and single nucleotide variation data, was obtained from TCGA database website (https://portal.gdc.cancer.gov/), totalizing 49 normal and 473 tumor samples. LUSC samples with no complete follow-up information were excluded to reduce the statistical bias. The copy number variation (CNV) data on TCGA-LUSC samples were collected from Xena functional genomics explorer (https://xenabrowser.net/).

### Identification of m6A-regulated immune LncRNAs in the smoking-associated TCGA-LUSC cohort

A total of 24 genes were found to act as, in light of previous studies, m6A regulators, including eight writers (*METTL3*, *METTL14*, *METTL16*, *WTAP*, *VIRMA*, *ZC3H13*, *RBM15*, and *RBM15B*), 14 readers (*YTHDC1*, *YTHDC2*, *YTHDF1*, *YTHDF2*, *YTHDF3*, *HNRNPC*, *FMR1*, *LRPPRC*, *HNRNPA2B1*, *IGF2BP1*, *IGF2BP2*, *IGF2BP3*, *RBMX*, and *EIF3A*), and two erasers (*FTO* and *ALKBH5*). For this reason, we extracted the expression matrix of the 24 m6A-related genes and lncRNAs in the smoking-associated TCGA-LUSC cohorts. The Pearson correlation coefficients were calculated to access the co-expression correlation between the 24 m6A-related genes and lncRNAs. LncRNAs that met the threshold of |Pearson correlation coefficients| > 0.30 and *p* < 0.001 were considered to be m6A-related lncRNAs. Furthermore, immune-related genes were obtained from IMMPORT (https://www.immport.org/home) and InnateDB (https://www.innatedb.ca/) websites. The co-expression relationship between the immune-related genes and lncRNAs was calculated by Pearson analysis, and lncRNAs with |Pearson correlation coefficients| > 0.40 and *p* < 0.001 were regarded as immune-related lncRNAs. Intersections of the two lncRNA sets were defined as m6A-regulated immune lncRNAs.

### Unsupervised consensus clustering analysis

To identify the molecular subgroups that were mediated by the expression patterns of m6A-related immune lncRNAs or DEGs in the smoking-associated LUSC cohort, the “ConsensusClusterPlus” package of R software (version 3.6.2) was utilized for unsupervised consensus clustering analysis (50 repetitions and 0.8 pItem). The cumulative distribution function (CDF) curve and consensus matrix were combined to find the most suitable k value that could provide a more representative clustering model for LUSC patients. The differences in gene expressions, clinical characteristics (age, gender, TNM stage, T, N, and smoking history), and prognosis between the classified subgroups were evaluated using the Wilcoxon test, chi-squared test, and Kaplan–Meier survival curves, respectively.

### Tumor microenvironment analysis

To evaluate the degree of infiltration of immune cells in different subgroups, we employed the ESTIMATE method to calculate the immune and stromal scores and estimate scores for each patient in the smoking-associated LUSC cohort with the help of “estimate” R package. After the risk score model was constructed, several common algorithms like EPIC, TIMER, MCP-counter, and XCELL were performed to compare the differences of infiltrating levels of immune cells between the high- and low-risk groups. The immune infiltration data on the TCGA-LUSC cohort were downloaded from the TIMER2.0 database (http://timer.cistrome.org/). In addition, we also estimated the expression of a series of immune checkpoint molecules and IFN-related genes using the Wilcoxon test in different subgroups as a necessary supplement for the immune cell infiltration analysis. The “maftools” R package was used to describe the mutation information of the top 20 genes with the highest mutation frequency in high- and low-risk groups.

### Functional enrichment analysis for DEGs between different clusters

The DEGs between the classified clusters were identified by means of the “limma” R package (adjusted *p*-value < 0.001), and the expression profile of these DEGs was then extracted from previously downloaded RNA-seq data. To further explore the involved biological processes and signal pathways of modules that were most relevant to risk scores, Gene Ontology (GO) and Kyoto Encyclopedia of Genes and Genomes (KEGG) analyses were carried out utilizing the “clusterProfiler” package of R software with the *p*-value < 0.05 as the significant threshold.

### Hub gene identification

The protein–protein interaction (PPI) network of these prognostic DEGs, which were identified by univariate Cox regression analysis, was constructed with the help of the STRING database (https://string-db.org/). Then, the network was enrolled in Cytoscape software (version 3.7.2), and cytoHubba plug-in was downloaded to calculate the top 10 hub genes by means of the “Degree” algorithm.

### Construction of the risk score model in the smoking-associated TCGA-LUSC cohort

By virtue of the previously identified m6A-related lncRNAs, a risk score model was constructed as follows. First, univariate Cox regression analysis was utilized to screen lncRNAs that were significantly associated with the overall survival (OS) of patients from the smoking-associated TCGA-LUSC cohort. Then, lncRNAs with the *p*-value < 0.05 in the univariate Cox regression analysis were further subjected to multivariate Cox regression analysis to identify the ones that could independently affect the prognosis of LUSC patients. Finally, a 12-lncRNA signature model for prognostic prediction in this cohort was successfully established based on the expression levels and corresponding coefficients of the selected lncRNAs. The risk score of each patient in the cohort was calculated by the following equation: risk score = $\overset{12}{\underset{i=1}{\sum }}\left(\beta i\text{∗}Expi\right)$ . In this formula, βi meant the coefficient derived from multivariate Cox regression, and Expi referred to the expression levels of the 12 lncRNAs in this model. Furthermore, all samples in the cohort were assigned to high- or low-risk groups according to their risk score with the median value as the cutoff value.

### Survival analysis and evaluation of the risk model

As the patients in the smoking-associated TCGA-LUSC cohort have been divided into high- and low-risk groups, we drew the Kaplan–Meier survival curves of the two groups with the help of “survival” and “survMiner” packages of R software, and the log-rank test was used to evaluate the survival difference between the two groups with *p* < 0.05 as the significant threshold. To access the exactitude of the risk model we have built, the receiver operating characteristic (ROC) curve was displayed and the area under the curve (AUC) was calculated by means of the “timeROC” R package. In addition, the “timeROC” package and “compare” function were utilized to contrast the AUC of the risk score with other clinical features at 1 year (*p* < 0.05 as significant).

### Independence assessment and stratification analysis of the risk model

The clinical characteristics and expression profiles of the 12 lncRNAs in high- and low-risk groups were exhibited as a heat map by virtue of the “pheatmap” R package. In addition, to make sure that the risk score of LUSC patients, which was calculated by the 12 lncRNA-related signature in this study, could act as an independent prognostic factor when compared to other traditional clinical features, univariate and multivariate Cox regression analyses were successively performed using OS as the dependent variable, while five potential prognostic factors, namely, smoking history, age, gender, TNM stage, and risk score, were incorporated in Cox regression analyses as independent variables. Furthermore, based on the results of multivariate Cox regression analysis, stratification analysis was carried out to further investigate whether the risk model would also have prognostic efficiency under the stratified clinical features that had been proven to be significantly associated with OS and acted as an independent prognostic factor for LUSC patients in this cohort. Moreover, for the classified high- and low-risk groups, gene set enrichment analysis (GSEA), which could provide a better assistance in searching for the potential downstream pathways and investigating the possible mechanisms underlying the association between risk score and prognosis, was performed by means of GSEA 4.0.3 software.

### Building and assessment of the nomogram

Subsequently, a nomogram was successfully developed using the independent risk factors derived from the multivariate Cox regression analysis in this cohort. The survival possibility at 1, 3, and 5 years from diagnosis of a given LUSC patient could be concisely predicted by putting these concerned factors independently affecting patients’ survival into this comprehensive scoring system. In addition, in order to assess the specificity, accuracy, and discriminative ability of the established nomogram, we drew a calibration curve with those three time points by making use of the “rms” package of R software.

### Drug sensitivity analysis

To provide references for the treatment of LUSC patients based on our established risk scoring system, we assessed the 50% inhibitive concentration (IC50) of a series of common chemotherapeutic drugs for patients in low- and high-risk groups using “pRRophetic” R package. *p* < 0.05 was considered to indicate significant differences between the two groups.

## Results

### Expression and mutation features of 24 m6A regulators in the smoking-associated LUSC cohort

**FIGURE 1:**
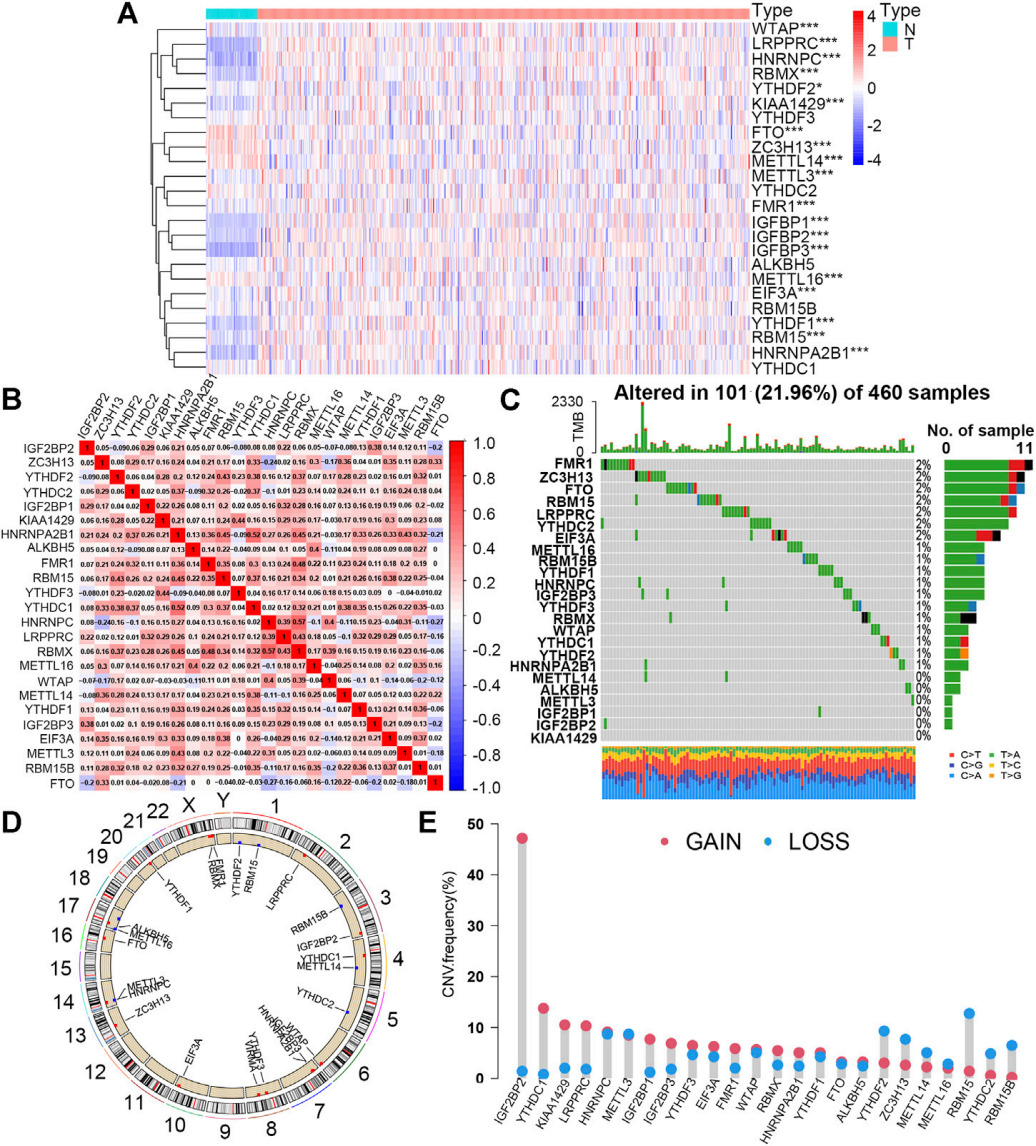
Expression levels and mutation features of 24 m6A regulators in the smoking-associated LUSC cohort from TCGA database. **(A)** Expression differences of 24 m6A regulators between normal (N) (n = 49) and tumor (T) (n = 473) samples (*p < 0.05; ***p < 0.001). **(B)** Expression correlation analysis of 24 m6A regulators in the smoking-associated LUSC cohort. Red and blue refer to positive and negative correlation, respectively. **(C)** Somatic mutation types and frequency of 24 m6A regulators in 460 LUSC samples with smoking history. **(D)** Locations and CNV types of 24 m6A genes on 24 different chromosomes. Red and blue refer to the increased and decreased CNV types, respectively. **(E)** CNV frequency of 24 m6A regulators in the smoking-associated LUSC cohort. Red and blue refer to the increased and decreased CNV types, respectively.

The detailed workflow of the present study is shown in Supplementary Figure S1. A total of 24 m6A regulators were incorporated into this study, and the expression levels of most of these proteins between the normal (49) and tumor (473) sample groups showed significant differences. According to the heat map (Figure 1A), 15 m6A regulators (YTHDF2, FMR1, METTL3, EIF3A, WTAP, RBM15, VIRMA, IGF2BP2, RBMX, YTHDF1, IGF2BP1, HNRNPA2B1, IGF2BP3, HNRNPC, and LRPPRC) were upregulated, and only 4 regulators (METTL16, METTL14, FTO, and ZC3H13) were downregulated in tumor samples compared to controls. In addition, five proteins (YTHDC2, YTHDC1, YTHDF3, RBM15B, and ALKBH5) had no significant distinction between the two groups. This result demonstrated that m6A regulators might play a vital role in tumorigenesis and progression for LUSC patients with smoking history. Furthermore, we observed that more than 80% of m6A regulators displayed a positive correlation with each other, suggesting that these proteins would have a common mode of action in regulating this kind of epigenetic modification of RNA (Figure 1B).

For the study of mutation characteristics, we first explored the frequency and types of somatic mutations of the 24 m6A regulators. During this analysis, 101 (21.96%) of the 460 samples with mutation information were found to possess mutations and 18 (75%) out of the 24 m6A regulators were mutated with the frequency varying from 1% to 2% (Figure 1C). Then, we analyzed the CNV of the 24 m6A regulators in the LUSC cohort. The CNV of the 24 m6A regulators and their positions on different chromosomes were visualized as the circos plot (Figure 1D). Figure 1E shows that IGF2BP2, YTHDC1, KIAA1429 (VIRMA), and LRPPRC had the most notable increases in copy number alterations, while RBM15, RBM15B, YTHCD2, YTHDF2, and ZC3H13 owned the decreased CNV, which was basically consistent with the expression changes between normal and tumor sample groups. The expression levels of the 24 m6A regulators between the TP53 wild and mutated groups were further compared and nine of them (37.5%) showing higher levels in the TP53 mutated group than that in the wild group, implying that mutated TP53 might promote the expression of these m6A-related genes (Supplementary Figure S2A).

The prognostic values of the 24 m6A regulators in this LUSC cohort were estimated by univariate Cox regression analysis. However, none of them had a significant effect on the overall survival of patients, and the results suggested that m6A regulators might play a role *via* their downstream modulated lncRNAs (Supplementary Figure S2B).

### Identification of m6A-related immune lncRNAs

**FIGURE 2:**
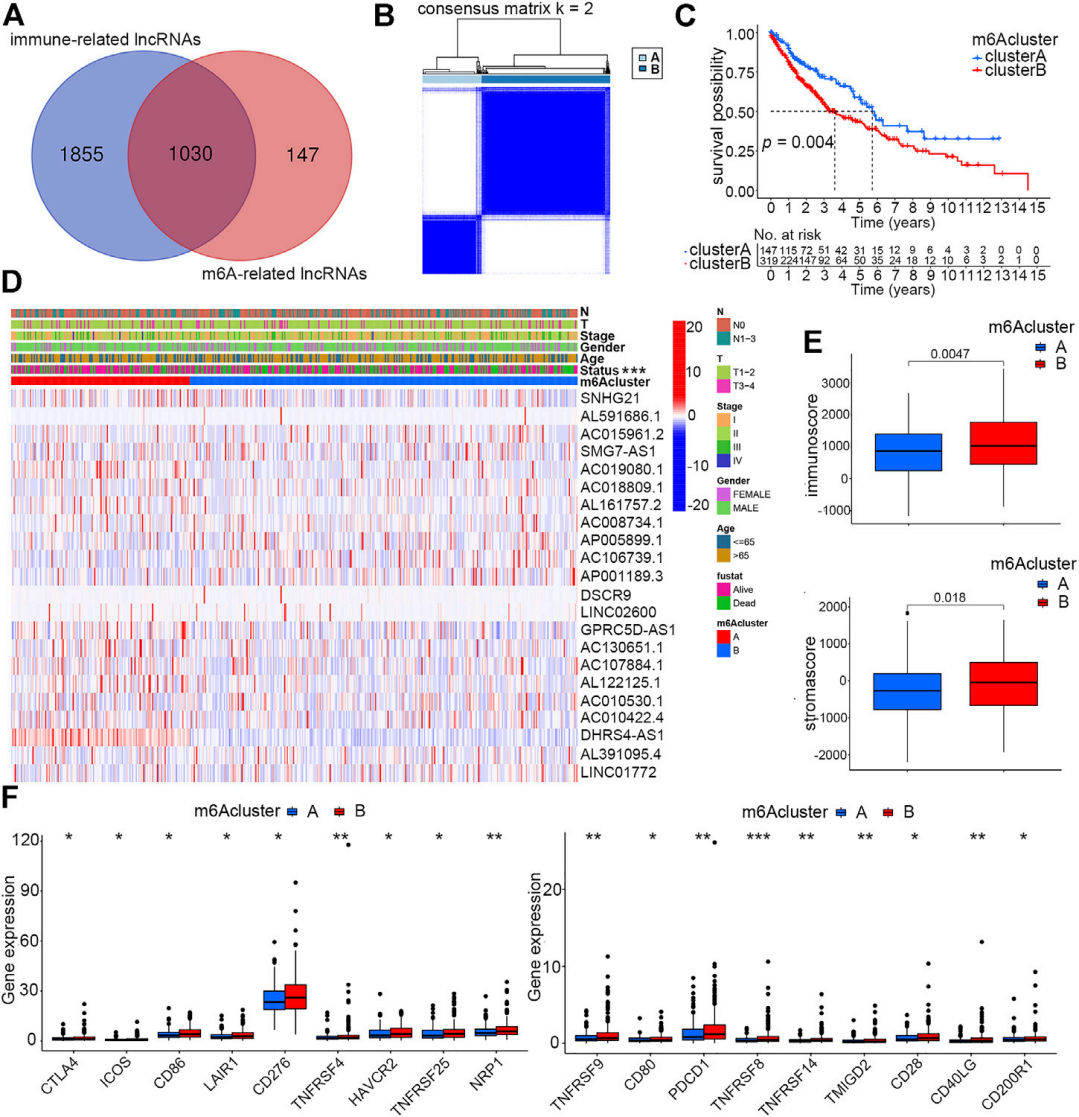
Identification of two distinct m6A clusters and differences of their clinical and immune features. **(A)** Identification of immune-related lncRNAs regulated by m6A regulators. **(B)** Two distinct m6A clusters were determined based on m6A-regulated immune-related lncRNAs *via* unsupervised consensus clustering analysis. **(C)** Overall survival differences between m6A clusters A and B by Kaplan–Meier survival analysis. Log-rank *p* = 0.004. **(D)** Differences of clinical features and expression profiles of m6A-regulated immune-related lncRNAs between clusters A and B (***p < 0.001) **(E)** Differences of immune and stroma scores by ESTIMATE analysis of smoking-associated LUSC samples between m6A clusters A and **B**. **(F)** Differences of expression levels of immune checkpoint molecules between m6A clusters A and B (*p < 0.05, **p < 0.01, and ***p < 0.001).

According to the criteria described previously, a total of 1,177 m6A-related lncRNAs and 2,885 immune-related lncRNAs were screened out. The intersection of the aforementioned two sets included 1,030 lncRNAs that were finally defined as m6A-related immune ones (Figure 2A). To further explore lncRNAs that could significantly affect the prognosis of smoking-associated LUSC patients, univariate Cox regression analysis was carried out, and 22 lncRNAs were identified as exhibiting prognostic values. Forest plot further revealed that eight of these prognostic lncRNAs were protective factors, with patients with higher expression exhibiting better prognosis (Supplementary Figure S3A). The expression profiles of 22 lncRNAs were extracted from RNA-seq data for further studies.

### Identification of m6A clusters based on m6A-related immune lncRNAs

To investigate whether the identified m6A-regulated immune-related lncRNAs could influence the prognosis and other clinical features of smoking-associated LUSC patients in a coordinated way, we performed unsupervised consensus clustering analysis by using the expression matrix of 22 prognostic lncRNAs previously identified by univariate Cox regression. According to the consensus matrix, we selected k = 2 as the optimal clustering parameter and divided these patients into two m6A clusters named cluster A (147 cases) and cluster B (319 cases) (Figure 2B). The Kaplan–Meier survival curve showed that the prognosis of patients in cluster A was significantly better than that in cluster B (Figure 2C). We further evaluated the differences in lncRNA expression levels and clinical features between the two clusters. According to the heatmap, expression levels of the 22 m6A-regulated immune-related lncRNAs were quite different between the two clusters. In addition, the survival status also showed a distinct distribution characteristic, which was consistent with the previous survival analysis (Figure 2D).

As the TME could directly or indirectly influence patient survival by regulating immune infiltration, our study further concentrated on investigating whether immune-related factors play a crucial role in causing different clinical performance of the two clusters. ESTIMATE analysis suggested that patients in cluster B had higher immune and stromal scores than patients in cluster A (Figure 2E). Much research has reported that high infiltration of immune cells, especially adaptive immune cells like activated CD4^+^ T cells and CD8^+^ T cells, could effectively promote the ability of the immune system of eliminating tumor cells, thereby inhibiting tumor growth (Tekpli et al., 2019). In this study, we speculated that it was the high stromal cell infiltration that counteracted the anti-cancer effects of immune cells, accounting for the reasons why patients in cluster B with higher immune scores exhibited poorer prognosis. Another factor directly affecting patient’s prognosis and sensitivity to immunotherapy is immune checkpoint proteins. We evaluated the expression levels of 18 immune checkpoint molecules between the two clusters, and the result showed that all these proteins, including PDCD1 and CTLA4, had higher expression in cluster B patients (Figure 2F).

### Identification of gene clusters based on DEGs

**FIGURE 3:**
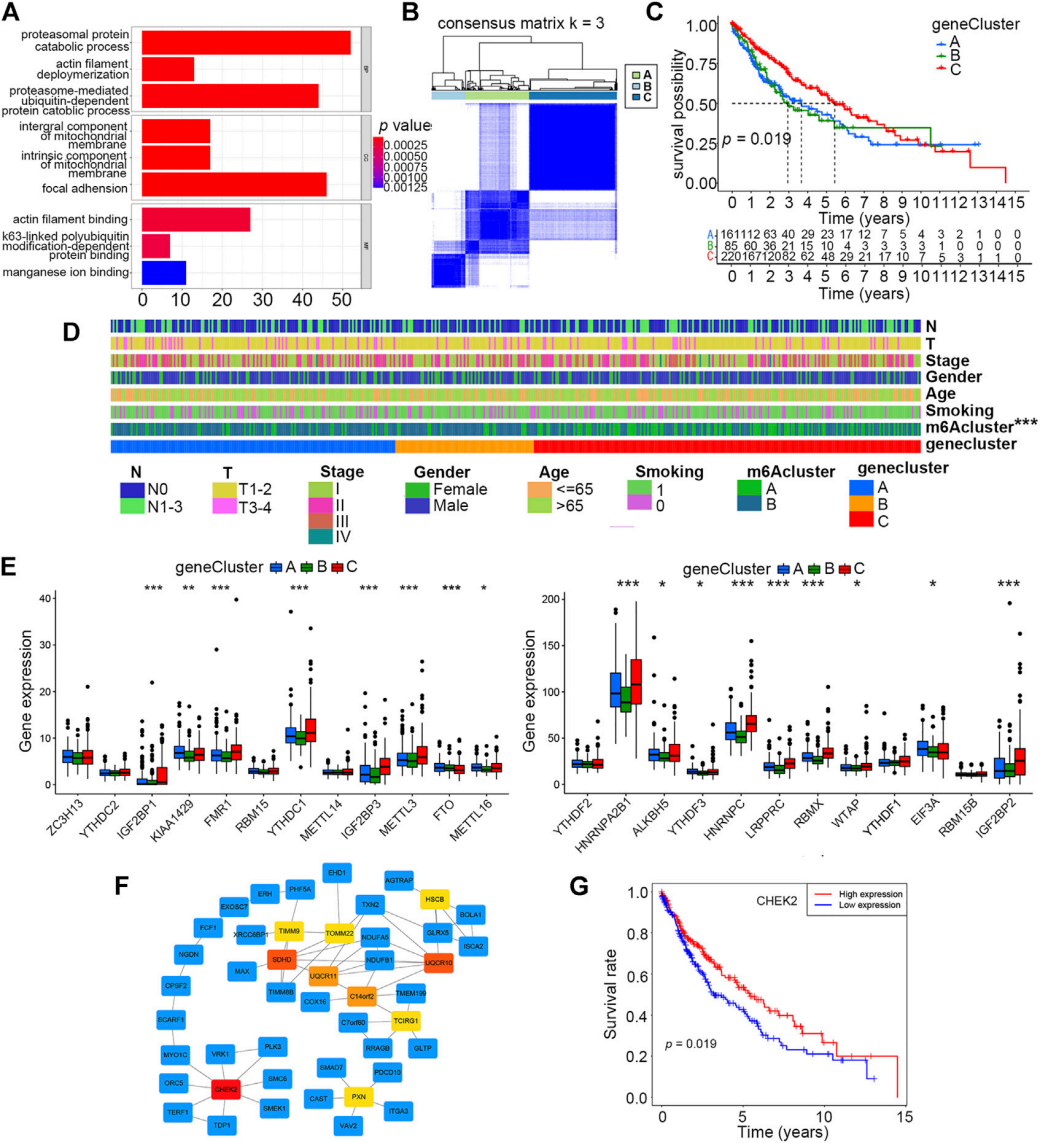
Identification of three distinct gene clusters based on DEGs of m6A clusters A and **B**. **(A)** Enrichment analysis of 1814 DEGs of m6A clusters A and B by GO. **(B)** Identification of three distinct gene clusters based on 230 prognostic DEGs of m6A clusters A and B *via* unsupervised consensus clustering analysis. **(C)** Overall survival differences among m6A clusters A, B, and C by Kaplan–Meier survival analysis. Log-rank *p* = 0.019. **(D)** Differences in clinical characteristics and m6A clusters among gene clusters A, B, and **C**. **(E)** Differences in expression levels of 24 m6A regulators among gene clusters A, B, and C (*p < 0.05, **p < 0.01, and ***p < 0.001). **(F)** Hub gene identification among DEGs by Cytoscape software and the Degree algorithm. The top 10 hub genes are marked from pale yellow to deep red, and the darker the color, the more important it is. The other related genes are labeled blue. **(G)** Overall survival differences between high and low expression levels of CHEK2 in the smoking-associated LUSC cohort by Kaplan–Meier analysis. Log-rank *p* = 0.019.

As the aforementioned categorized clusters have been evidenced to be closely associated with patient prognosis, we further explored the potential mechanisms by functional enrichment analysis using DEGs between the two clusters. By means of the “limma” R package, a total of 1814 DEGs were identified, and their expression profiles combined with relevant clinical information were extracted from original data. GO analysis revealed that DEGs were more likely to be enriched in adhesion- and metabolism-related processes or pathways, which were previously deemed to be closely correlated with tumor progression (Figure 3A).

To get a better understanding of how these DEGs could have an impact on prognosis, we conducted unsupervised consensus clustering analysis based on the expression profiles of 230 prognostic DEGs singled out by univariate Cox regression, and three distinct gene clusters, A, B, and C, were finally identified (Figure 3B). The prognosis of patients in cluster C was better than that of those in clusters A and B, according to the Kaplan–Meier survival curve (Figure 3C). Furthermore, the distributions of clinical characteristics, including the m6A cluster, among these three clusters were further analyzed. The result showed that the m6A cluster, but not other clinical characteristics, differed among these three gene clusters (Figure 3D). By evaluating the expression levels of the 24 m6A regulators, we found that 17 of them (70.8%) were differentially expressed among the three gene clusters, and most of these differentially expressed regulators had higher expression levels in cluster C than that in the other two clusters (Figure 3E).

### Hub gene identification

The top 10 hub genes among these DEGs were identified by means of Cytoscape software, and CHEK2 was ranked as the most important one using the “Degree” algorithm (Figure 3F). We further explored the prognostic value of CHEK2 in this smoking-associated LUSC cohort, and the result showed that patients with a higher CHEK2 expression were more likely to exhibit better overall survival (Figure 3G).

### Establishment and validation of a risk score model

**FIGURE 4:**
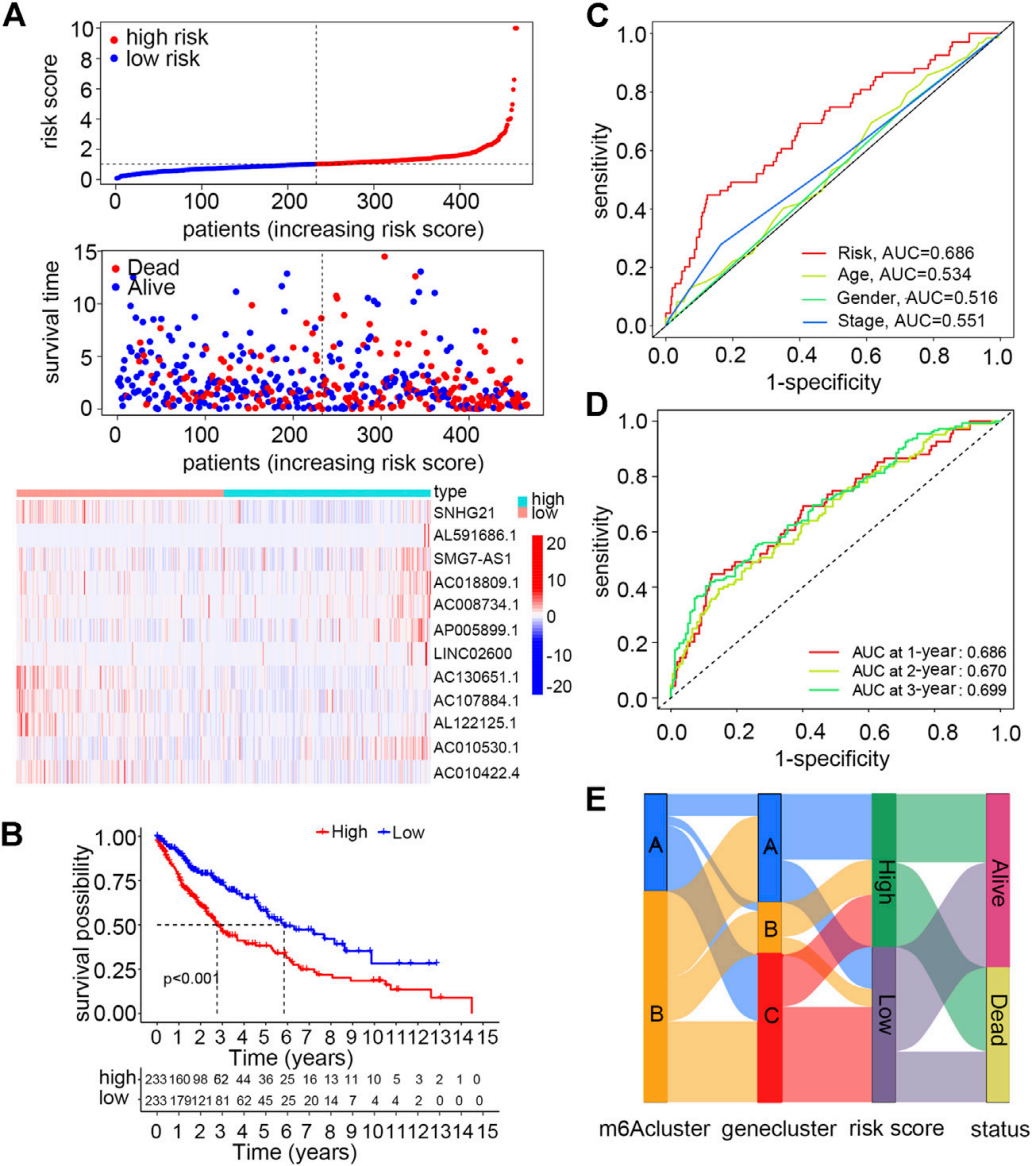
Construction and validation of a risk model using m6A-regulated immune-related lncRNAs for smoking-associated LUSC patients. **(A)** Distribution of the risk score and survival status and the expression levels of m6A-regulated immune-related lncRNAs between high- and low-risk groups. **(B)** Overall survival differences between high- and low-risk groups by Kaplan–Meier analysis. Log-rank *p* < 0.001 **(C)** ROC curves and AUC values of the risk score to compare the sensitivity and specificity of the risk score and other clinical features at 1 year. **(D)** ROC curves and AUC values of the risk score at 1, 2, and 3 years. **(E)** Sankey diagram of patients in distinct m6A clusters corresponding to different gene clusters, risk groups, and survival status.

As described earlier, 22 of these m6A-related lncRNAs in the smoking-associated TCGA-LUSC cohort were significantly associated with survival prognosis. Subsequently, multivariate Cox regression analysis was performed for these prognosis-related lncRNAs. As a result, 12 lncRNAs were selected to construct a risk signature for LUSC patients. The risk score of each patient could be calculated by the linear combination of the expression value of 12 lncRNAs weighted by their coefficients as follows: risk score = (−0.2264 × expression value of SNHG21) + (0.0458 × expression value of AL591686.1) + (0.8498 × expression value of SMG7-AS1) + (0.4858 × expression value of AC018809.1) + (1.3643 × expression value of AC008734.1) + (0.3552 × expression value of AP005899.1) + (0.1272 × expression value of LINC02600) + (−1.3126 × expression value of AC130651.1) + (−0.7355 × expression value of AC107884.1) + (−0.3529 × expression value of AL122125.1) + (1.2541 × expression value of AC010530.1) + (−0.7161 × expression value of AC010422.4). According to the formula, AL591686.1, SMG7-AS1, AC018809.1, AC008734.1, AP005899.1, AC010530.1, and LINC02600, which exhibited positive coefficients in the risk signature, would predict a poor prognostic ending for those with high expression levels of these lncRNAs. Meanwhile, the other lncRNAs with negative coefficients were regarded as protective factors and higher expression levels of these lncRNAs often indicated a longer overall survival. Patients were assigned to high- or low-risk groups based on the median value of risk scores. According to the risk plot, the risk score model could effectively separate patients with unique survival status into different risk groups, in which the high-risk group had more deceased patients than the low-risk group. Moreover, seven lncRNAs featured with positive coefficients had low expression levels in high-risk group patients, while the remaining five lncRNAs showed opposite expression patterns (Figure 4A). The Kaplan–Meier survival curve suggested that the overall survival of patients in the high-risk group was significantly worse than those in the low-risk group (*p*-value < 0.05) (Figure 4B). In order to validate the specificity and sensitivity of the established risk model, we depicted the ROC curve and calculated the AUC under different conditions. Figure 4C shows that the risk score had a higher AUC value than other clinical features like age, gender, and TNM stage at 1 year (*p*-value < 0.05), indicating that this scoring system was superior to others in assessing the prognosis of patients. Moreover, the AUC values under 1, 2 and 3 years were 0.686, 0.670, and 0.699, respectively (Figure 4D). Finally, a Sankey diagram was drawn to describe the relationship of patients in distinct m6A clusters with gene clusters, risk scores, and survival status (Figure 4E).

To have a better understanding of the expression features of the 12 lncRNAs involved in the risk model and their correlation with m6A regulators, we first evaluated their expression levels between normal and tumor sample groups, and all of them were abnormally expressed according to the heatmap (Supplementary Figure S3B). Further analysis demonstrated that there were significant correlations between m6A regulators and the expression of 12 lncRNAs (Supplementary Figure S3C). Another Sankey diagram was depicted to clarify the relationship of the 12 lncRNAs with distinct m6A regulators and risk types (Supplementary Figure S3D).

### Independent prognostic and stratified analysis of the risk model

**FIGURE 5:**
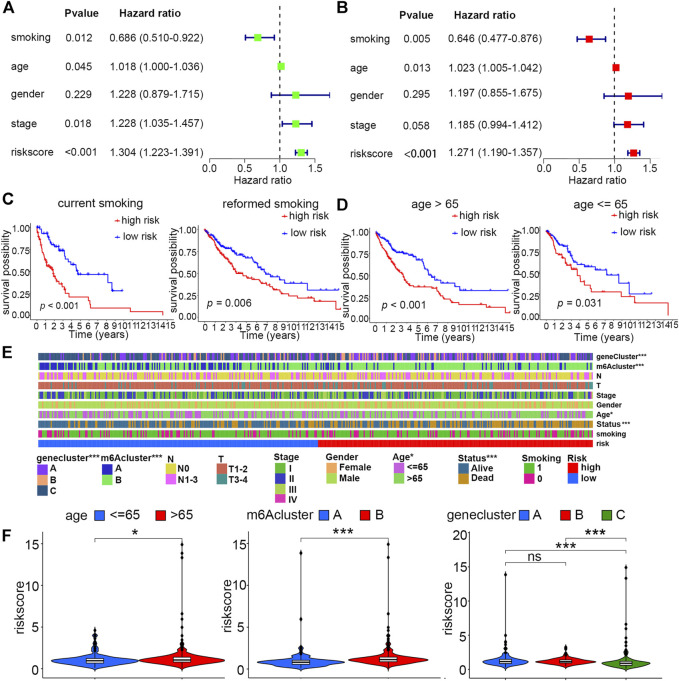
Independent prognostic and stratified analyses of the risk model. **(A)** Univariate and **(B)** multivariate Cox regression analyses of risk score and other clinical features. **(C)** Kaplan–Meier curves of high- and low-risk groups with stratified smoking history. **(D)** Kaplan–Meier curves of high- and low-risk groups with stratified age. **(E)** Differences in clinical characteristics, m6A clusters, and gene clusters between high- and low-risk groups (*p < 0.05; ***p < 0.001) **(F)** Distribution of the risk score between age, m6A cluster, and gene cluster subgroups (*p < 0.05; ***p < 0.001).

For the sake of evaluating whether the constructed risk scoring system could predict the prognosis as an independent factor, we performed univariate and multivariate Cox regression analyses by putting risk scores together with other clinical features, including smoking history (reformed smoking vs. current smoking), age (≤ 65 years vs. > 65 years), gender (female vs. male), and TNM stage (I vs. II vs. III vs. IV) into each analysis. The result suggested that risk score, age and smoking history, but not gender and TNM stage, are risk factors that could independently affect patient prognosis (Figures 5A,B). Furthermore, for age and smoking status, which have been proven to be independent risk factors *via* multivariate Cox regression analysis, we performed stratified analysis by dividing them into two diverse subgroups. Patients with high scores under all these stratified conditions, including age (≤ 65 and >65 years old) and smoking history (reformed smoking and current smoking), presented poorer prognosis than low-score patients (Figures 5C,D).

For the constructed risk score model, we explored the relationship of risk scores with two different kinds of categorized clusters and clinical features, including smoking history, age, gender, TNM stage, T, N, and survival status. Among these factors, the m6A cluster, gene cluster, survival status, and age were distributed differently between low- and high-risk groups (Figure 5E). In detail, patients with features of m6A cluster A, gene cluster C, and age ≤ 65 years old were more likely to exhibit lower risk scores (Figure 5F).

### GSEA analysis of the risk score groups

GSEA analysis was performed to explore potentially activated pathways in low- and high-risk groups. In the high-risk group, we found many enriched immune-related pathways such as “antigen processing and presentation,” “cytokine–cytokine receptor interaction,” “leukocyte transendothelial migration,” “chemokine signaling pathway,” “natural killer cell-mediated cytotoxicity,” and “toll-like receptor signaling pathway.” However, there were only five pathways that were enriched in the low-risk group, like “basal transcription factors” and “RNA degradation” (Supplementary Figure S4).

### Correlation analysis of risk scores with immune infiltration and immunotherapy

**FIGURE 6:**
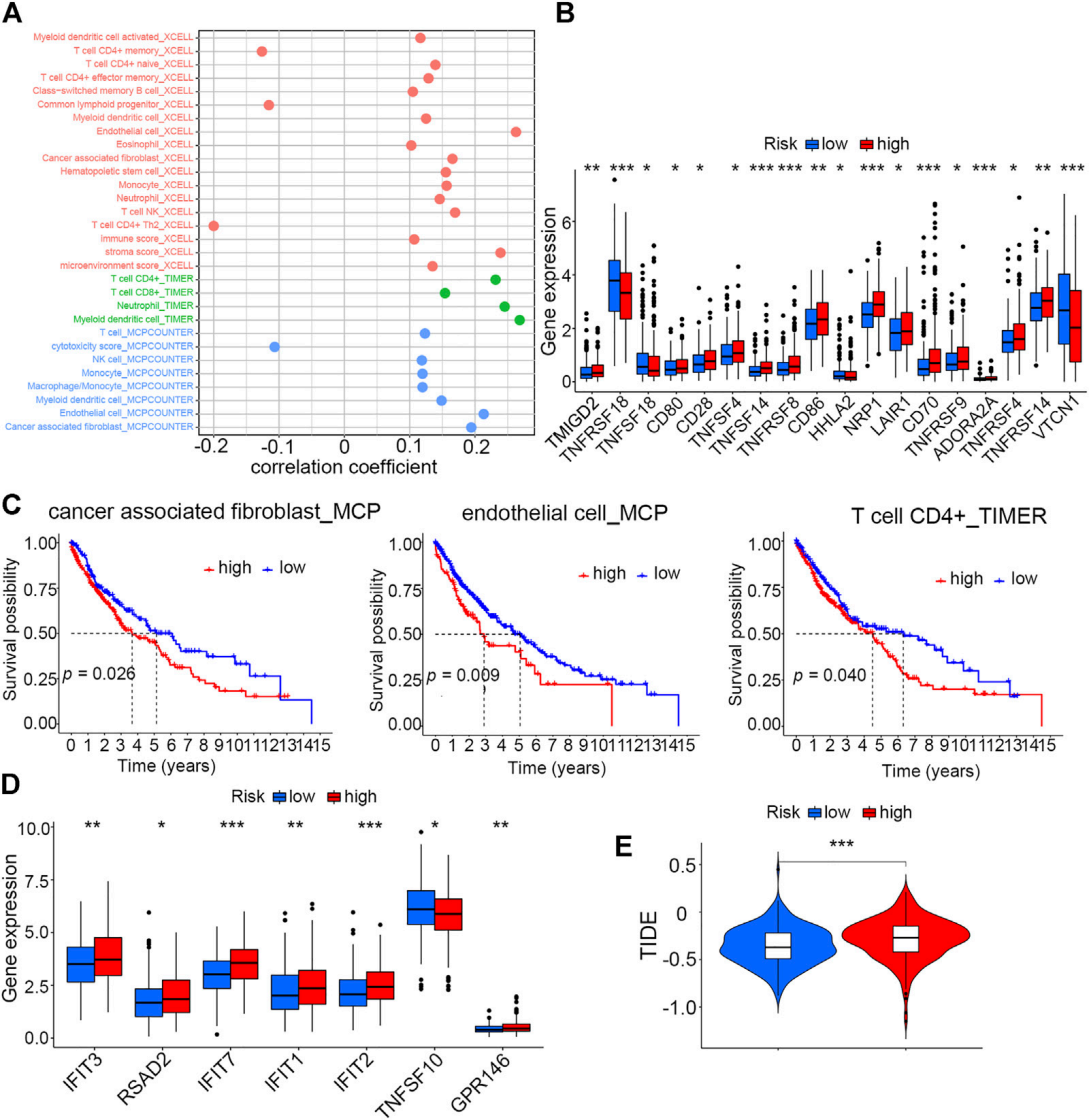
Correlation analysis of risk scores with the TME and immunotherapy **(A)** Correlation analysis of the risk score with infiltration levels of immune cells by three different algorithms. **(B)** Differences in expression levels of immune checkpoint molecules between high- and low-risk groups (*p < 0.05, **p < 0.01, and ***p < 0.001). **(C)** Kaplan–Meier analysis of cancer-associated fibroblasts, endothelial cells (MCP), and CD4^+^ T cells (TIMER) between high- and low-risk groups. **(D)** Differences in expression levels of seven IFN-related genes between high- and low-risk groups (*p < 0.05, **p < 0.01, and ***p < 0.001). **(E)** Tumor immune dysfunction and exclusion (TIDE) analysis to predict the sensitivity of patients in high- and low-risk groups to immunotherapy (***p < 0.001).

Three different algorithms, including TIMER, xCell, and MCP-counter, were utilized to assess and compare the immune infiltration levels in high- and low-risk groups. These algorithms suggested that the infiltration levels of immune cells like CD4^+^ T cells, CD8^+^ T cells, NK cells, and macrophage cells, as well as some kinds of stromal cells such as endothelial cells and cancer-associated fibroblasts, were positively correlated with risk scores (Figure 6A). Moreover, an analysis aiming to explore the correlation of risk scores with immune checkpoints was performed, and the result showed that patients in the high-risk score group had elevated expression levels of most of these important biomarkers in spite of no significant expression difference of PDL1 between the two groups (Figure 6B, Supplementary Figure S5A). Then, we evaluated the survival prognosis of patients under the different infiltration levels of stromal and immune T cells. The high infiltration levels of both the endothelial cells and cancer-associated fibroblasts using MCP-counter and xCell algorithms indicated poor prognosis (Figure 6C, Supplementary Figure S5B). However, patients with high CD4^+^ T-cell infiltration had worse overall survival than those with low infiltration (Figure 6C). As interferon (IFN) has been proven to be crucial in regulating tumor progression, we compared the expression levels of seven IFN-related genes between the high- and low-risk groups. Most of these IFN-related genes showed higher levels in the high-risk group than in the low-risk group (Figure 6D). In addition, the mutation information of the top 20 genes with the highest alteration frequencies in the two risk groups was visualized as the waterfall plot (Supplementary Figure S5C,D).

Given that the two risk groups had distinct immune characteristics, Tumor Immune Dysfunction and Exclusion (TIDE) was analyzed to compare the sensitivity of high- and low-score patients to immunotherapy. The result demonstrated that the high-risk group with higher TIDE scores was more likely to resist immunotherapy compared to the low-risk group (Figure 6E).

### Drug sensitivity analysis based on the risk score model

**FIGURE 7:**
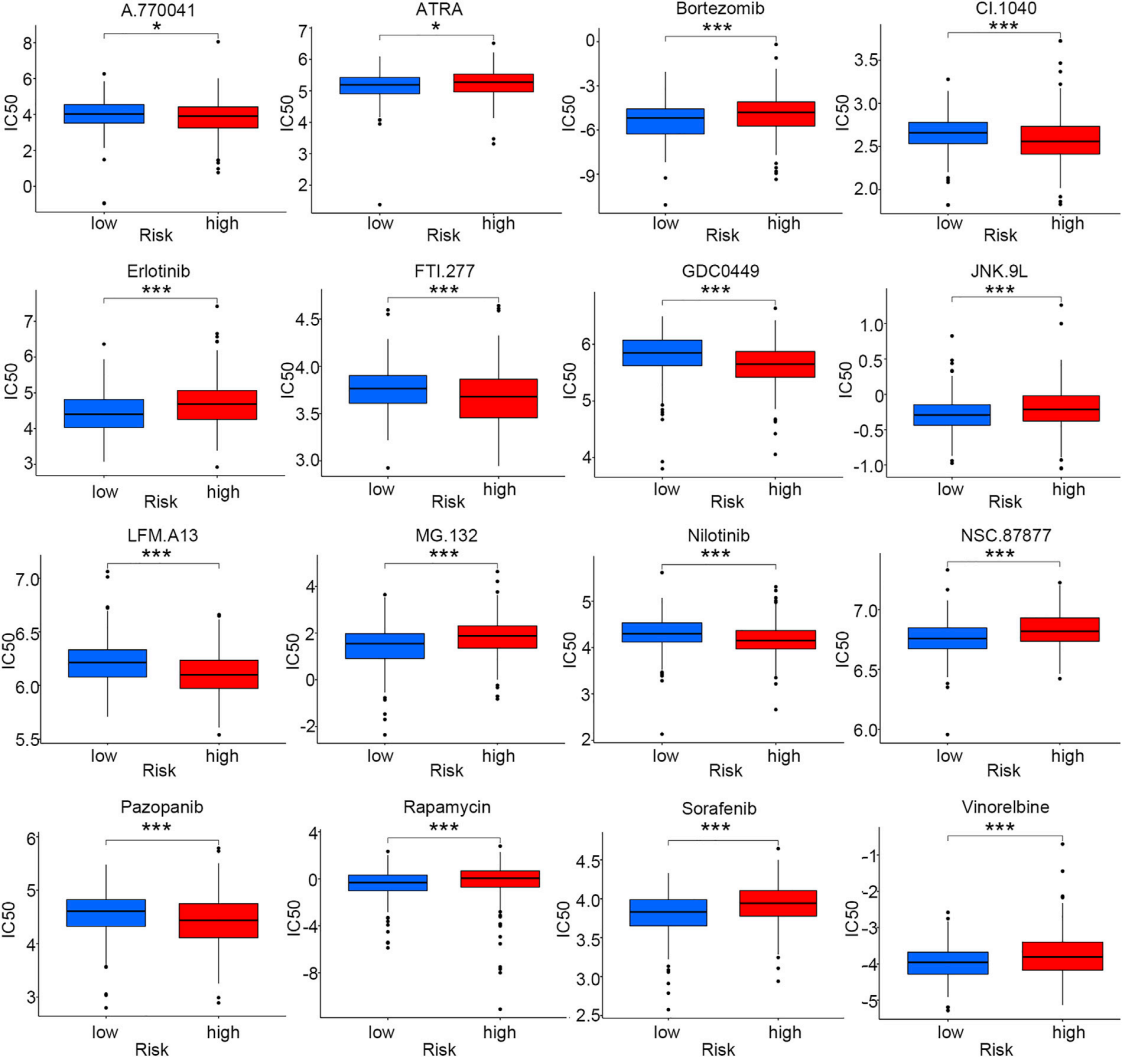
Drug sensitivity comparison for smoking-associated LUSC patients between low- and high-risk groups (*p < 0.05; ***p < 0.001).

As chemotherapy and targeted therapy have become the most important treatment for LUSC patients, identifying the subgroup of patients that was sensitive to specific drugs could promote their therapeutic effectiveness. Sixteen commonly used anti-tumor drugs were incorporated into this study to be evaluated in each risk group. According to Figure 7 **,** the low-risk group was more likely to respond to ATRA, bortezomib, erlotinib, JNK.9L, MG.132, NSC.87877, rapamycin, sorafenib, and vinorelbine, while the high-risk group was more sensitive to A.770041, CI.1040, FTI.277, GDC0449, LFM. A13, nilotinib, and pazopanib.

### Construction and evaluation of the nomogram

**FIGURE 8:**
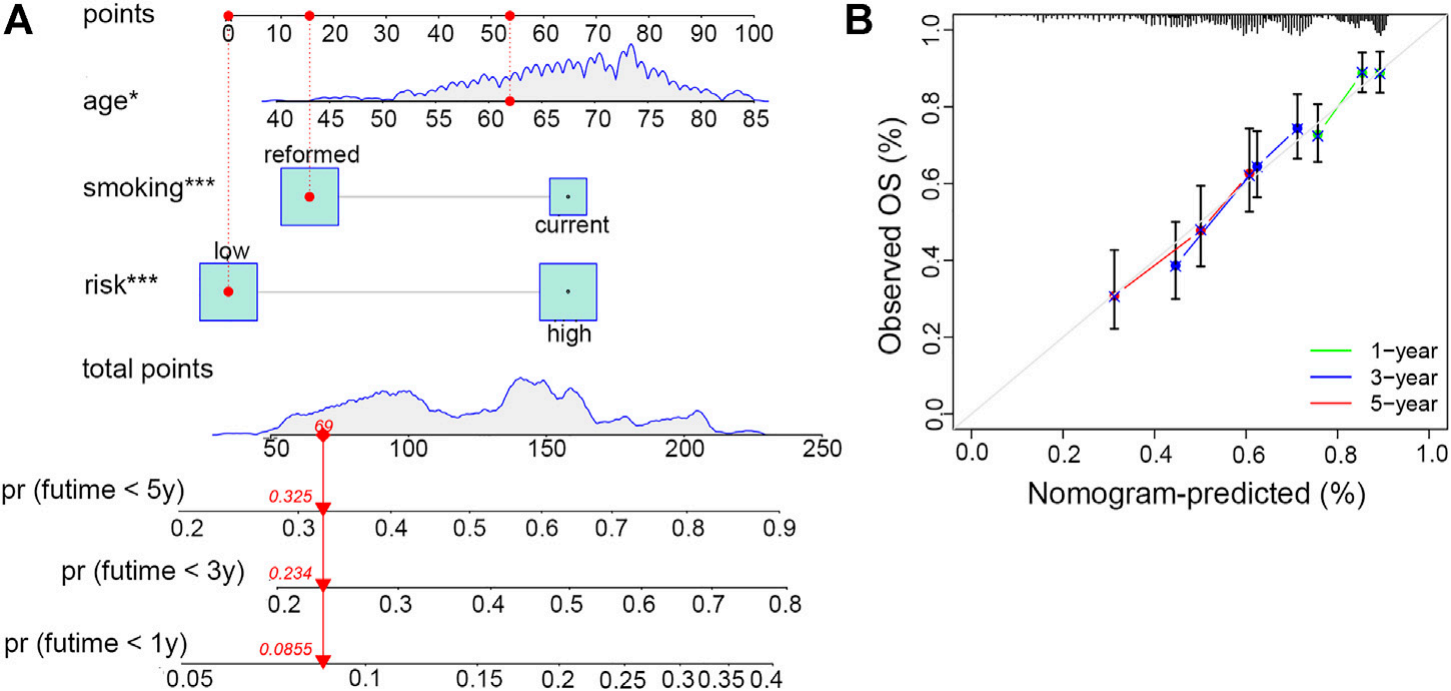
Establishment and evaluation of the clinical nomogram. **(A)** Nomogram to predict the survival possibility of smoking-associated LUSC patients at 1, 3, and 5 years. **(B)** Calibration curves to validate the prediction efficiency of the nomogram at 1, 3, and 5 years.

In order to conveniently predict the survival prognosis of LUSC patients, we constructed a nomogram by incorporating the risk score and two other prognostic risk factors, namely, age and smoking history, into this predictor (Figure 8A). By means of this nomogram, the survival possibility of a specific patient under 1, 3, and 5-years could be forecasted. The calibration curve revealed that the predicted overall survival of patients was basically the same as the observed outcome, indicating the high precision of this prediction model (Figure 8B).

## Discussion

m6A RNA methylation, which is considered to be the most common epigenetic modification in eukaryotic species, participates in the occurrence and progression of tumors in multiple ways (Jiang et al., 2021). In the present study, we first described the expression profiles and mutation features of 24 m6A regulators in LUSC patients with smoking history. As none of them could significantly affect the prognosis, we speculated that these regulators might act in a coordinated manner *via* modulating methylation levels on downstream RNA molecules, and the results were further verified by their expression correlation analysis. Until now, only one research concerning m6A regulators in LUSC has reported that the hypoxia-mediated high expression of YTHDF2, an important m6A reader, could predict a worse prognosis of LUSC, which was basically consistent with our conclusion (Xu et al., 2022).

To better understand whether m6A-regulated downstream lncRNAs could affect prognosis of patients with LUSC through immune regulatory mechanisms, we picked out m6A-regulated immune-related lncRNAs for further studies. Interestingly, 87.51% (1,030/1,177) of m6A-regulated lncRNAs were considered immune-related, which further validated our hypothesis that m6A RNA modification is deeply involved in immune-related processes. Accumulating evidence suggested that lncRNAs could directly or indirectly regulate the immune response and facilitate the occurrence of immune escape of cancer cells *via* a variety of mechanisms. Peng et al. (2022) have reported that lipopolysaccharides could effectively facilitate immune escape of hepatocellular carcinoma cells by regulating m6A modification on MIR155HG lncRNA to upregulate the PDL1 expression. However, the impact of m6A modification of lncRNAs on immune response in LUSC and the associated mechanisms have not yet been reported by experimental studies.

Unsupervised consensus clustering has become a routine method to divide samples into several typical molecular subgroups based on the expression levels of genes of interest. Gu et al. (2021) classified LUSC samples into different groups by directly incorporating m6A regulators into this clustering model. However, the research did not provide details on the differences of prognosis, immune landscape, and other information among those three distinct groups. In this study, we found that clusters categorized on the basis of m6A-regulated immune-related lncRNAs not only had different distributions of clinical outcomes and features but also represented diverse tumor microenvironment landscapes. Furthermore, we also demonstrated that m6A-regulated immune-related lncRNAs may have profound influence on clinical characteristics of LUSC patients, and the underlying mechanisms might involve changes in the tumor microenvironment. In particular, we also found that patients in the cluster featured with poor prognosis had high levels of immune and stromal scores and overexpressed immune checkpoint molecules. Thus, m6A-regulated immune-related lncRNAs could cooperate and be used as predictors of prognosis and immune response of patients with LUSC.

The risk model is a common method for predicting clinical outcomes of cancer patients. When previous studies concerning to LUSC directly brought m6A regulators into the model, the predictive effects were often unsatisfactory (Li and Zhan, 2020). In the present study, we established a risk model by means of 12 m6A-regulated immune-related lncRNAs selected by univariate and multivariate Cox regression analyses. In particular, all the lncRNA expression levels, clinical features, prognosis, TME landscapes, and drug sensitivities varied between the two risk groups. Thus, our risk model not only had a certain degree of clinical prediction significance but also provided guidance for personalized treatment in terms of immune-related mechanisms.

We noticed that the infiltration levels of CD4^+^ T cells, CD8^+^ T cells, NK cells, cancer-associated fibroblasts, and endothelial cells increased with the elevation of risk scores. Growing evidence has accumulated that CD4^+^ and CD8^+^ T cells are important in proinflammatory response and anticancer immunity, leading to a more favorable clinical outcome (Schneider et al., 2021). However, we did not observe such prognosis in patients with high risk scores because of the following possible reasons. First, most infiltrating T cells in tumors might stay in a dysfunctional state, and these “exhausted” T effectors may have lost their inhibitory control of cancers in spite of high infiltration levels (Chen and Mellman, 2017). Second, existing evidence indicated that the stroma cells, like cancer-associated fibroblasts, could directly interact with T cells, suppressing the immune response *via* immune checkpoint activation (Lakins et al., 2018).

Previous studies have categorized tumors into three different immune subtypes including the inflamed phenotype, immune-excluded phenotype, and immune-desert phenotype based on infiltration levels of immune cells and expression degrees of checkpoint proteins (Chen and Mellman, 2017). Although we could not draw a conclusion as to which kind of immune phenotype that the cluster or risk group belongs to, we could still get some enlightenment from this classification system for giving patients individual immunotherapy strategies on the basis of our risk model. According to the TIDE analysis, patients in the high-risk score group were more likely to resist the immunotherapy of checkpoint inhibitors. As only fresh “exhausted” T cells were partly sensitive to PD1/PDL1 inhibitors and “hyperexhausted” T cells might be totally unrecoverable under immunotherapies, PD1/PDL1 blockade combined with other methods that enhanced the efficiency of immunotherapy would be important for this group of patients. Moreover, according to the KEYNOTE-407 study, patients with advanced LUSC could still benefit from pembrolizumab combined with chemotherapy, regardless of positive or negative PDL1 expression (Zhao and Wang, 2020). Our study revealed that there was no significant difference in the PDL1 expression level between high- and low-risk groups.

There were some limitations to our research. First, we only included TCGA-LUSC cohort into the present study. As most expression profiles of LUSC patients published in the GEO database were derived from a microarray, we could not get all the expression data on lncRNAs. Thus, additional retrospective studies with complete lncRNA information and large-scale prospective studies are needed to validate the efficiency of our risk model. Second, as all the information on these prognostic lncRNAs involved in our risk model was obtained from a public database, it is necessary to determine their expression and mutation in newly collected clinical samples and further validate their biological functions through *in vitro* and *in vivo* experiments. Third, lncRNAs have been reported to be involved in tumor immunosuppression through multiple mechanisms and both endogenous and exosome-carried lncRNAs could play an important role in such a process. The present risk model incorporated 12 m6A-regulated immune-related lncRNAs and whether these prognostic lncRNAs could be specially targeted and thus reverse the suppressive TME need further research. Finally, the specific molecular mechanisms of how the risk model could predict the TME remain unclear and need further studies.

In conclusion, the present study comprehensively analyzed the value of m6A-regulated immune-related lncRNAs in predicting clinical features, prognosis, and the tumor microenvironment for LUSC patients with a clear smoking history. Thus, our findings could provide new ideas in giving better clinical decisions and personalized immunotherapy for these patients.

## Data Availability

The original contributions presented in the study are included in the article/Supplementary Material; further inquiries can be directed to the corresponding author.
