# Supplementary figures and images for: Comprehensive analysis of molecular features, prognostic values, and immune landscape association of m6A-regulated immune-related lncRNAs in smoking-associated lung squamous cell carcinoma
Source: Front Genet. 2022 Aug 10;13:887477. doi: 10.3389/fgene.2022.887477 (PMC9399351; doi:10.3389/fgene.2022.887477)

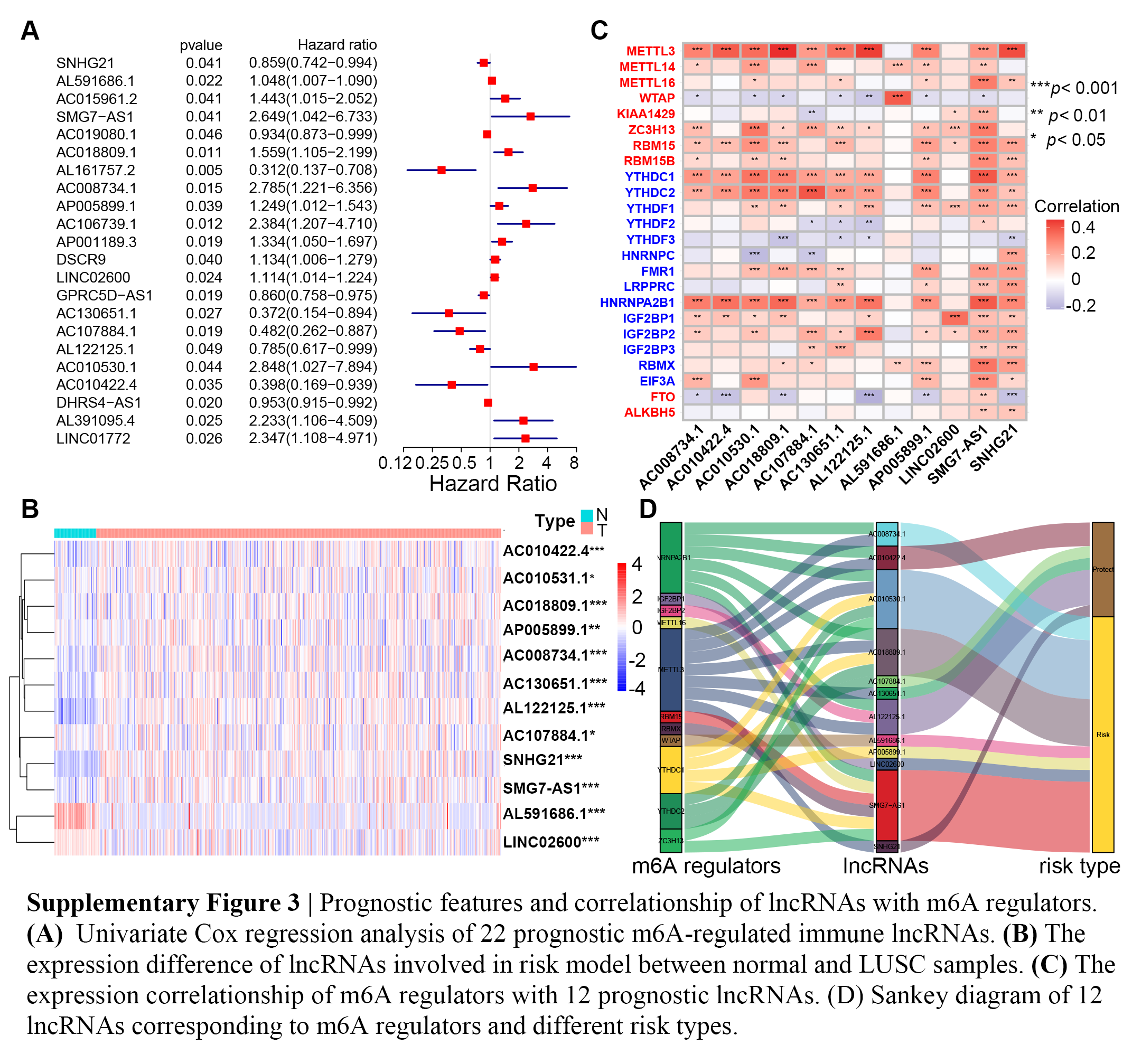

Supplement: Supplementary file 1 [file Image3.TIF]

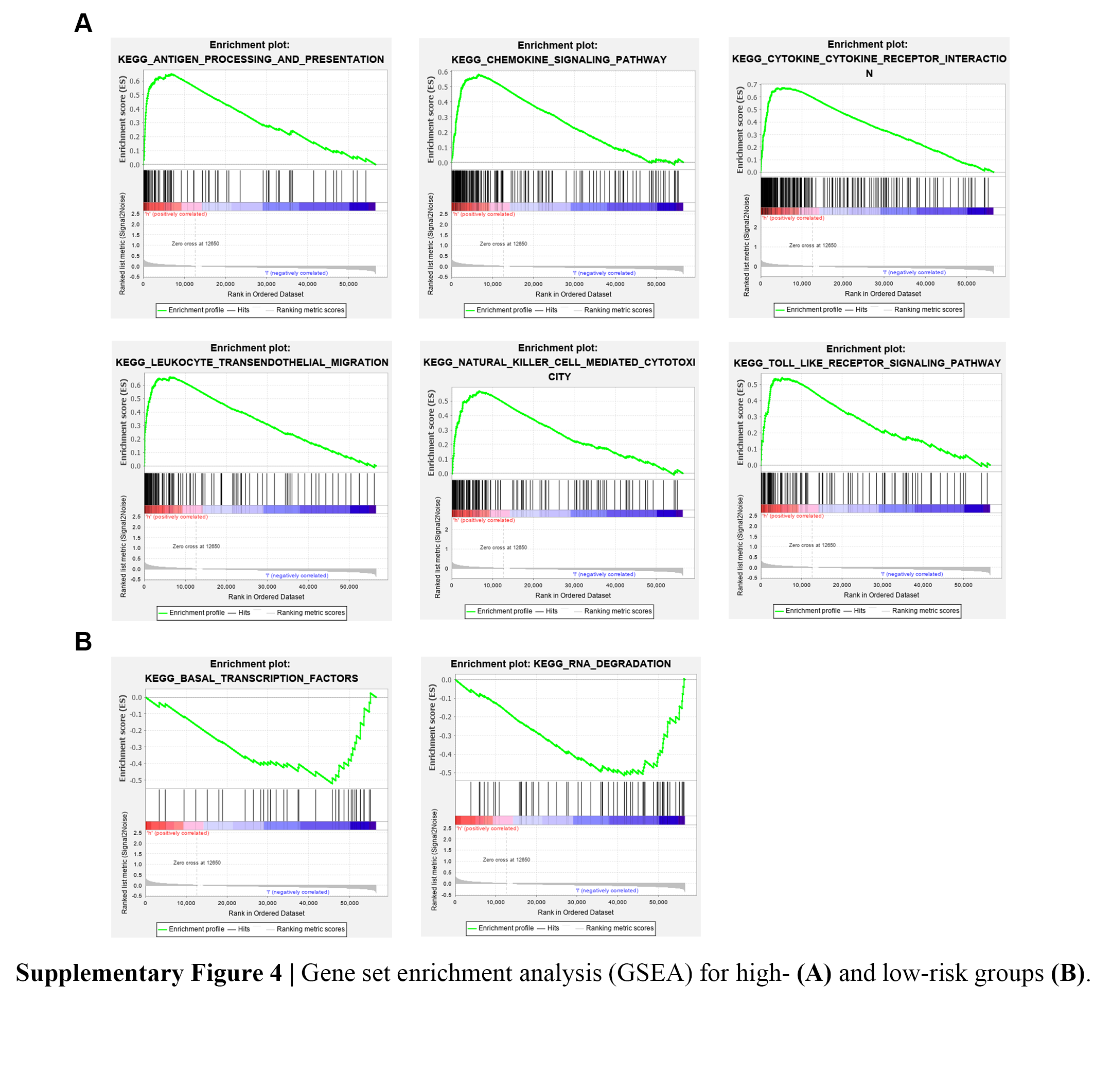

Supplement: Supplementary file 2 [file Image4.TIF]

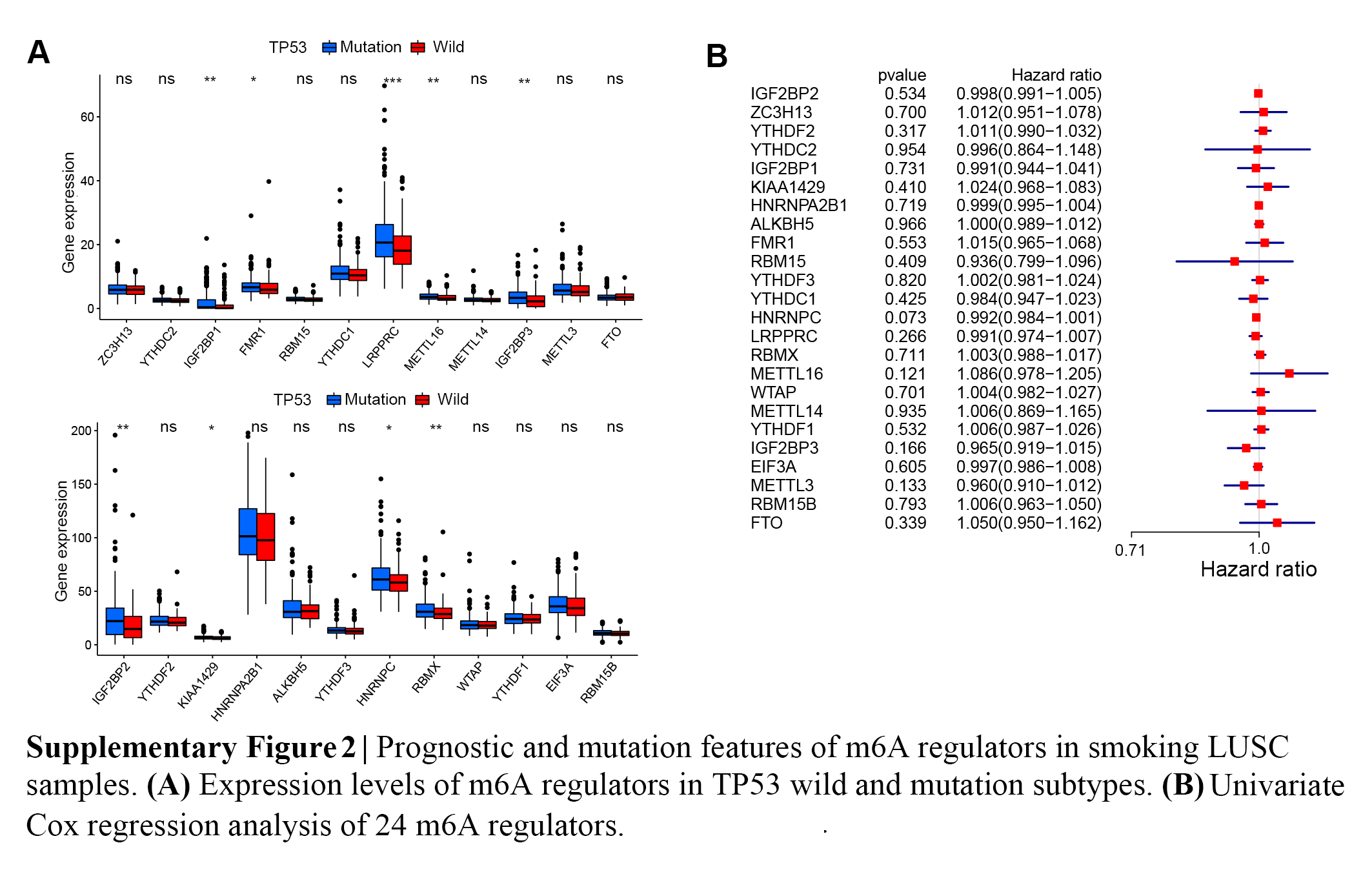

Supplement: Supplementary file 3 [file Image2.TIF]

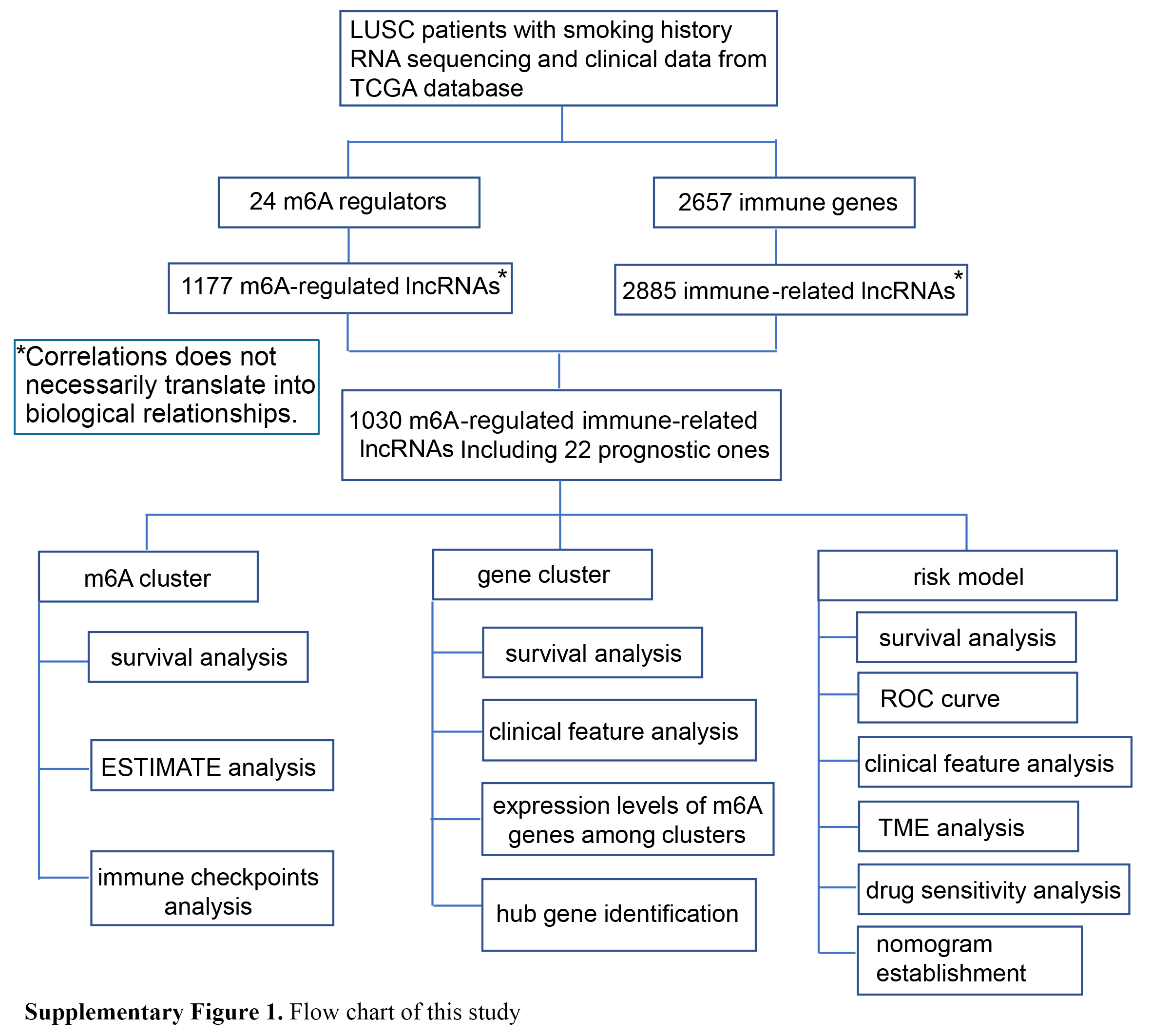

Supplement: Supplementary file 4 [file Image1.TIF]

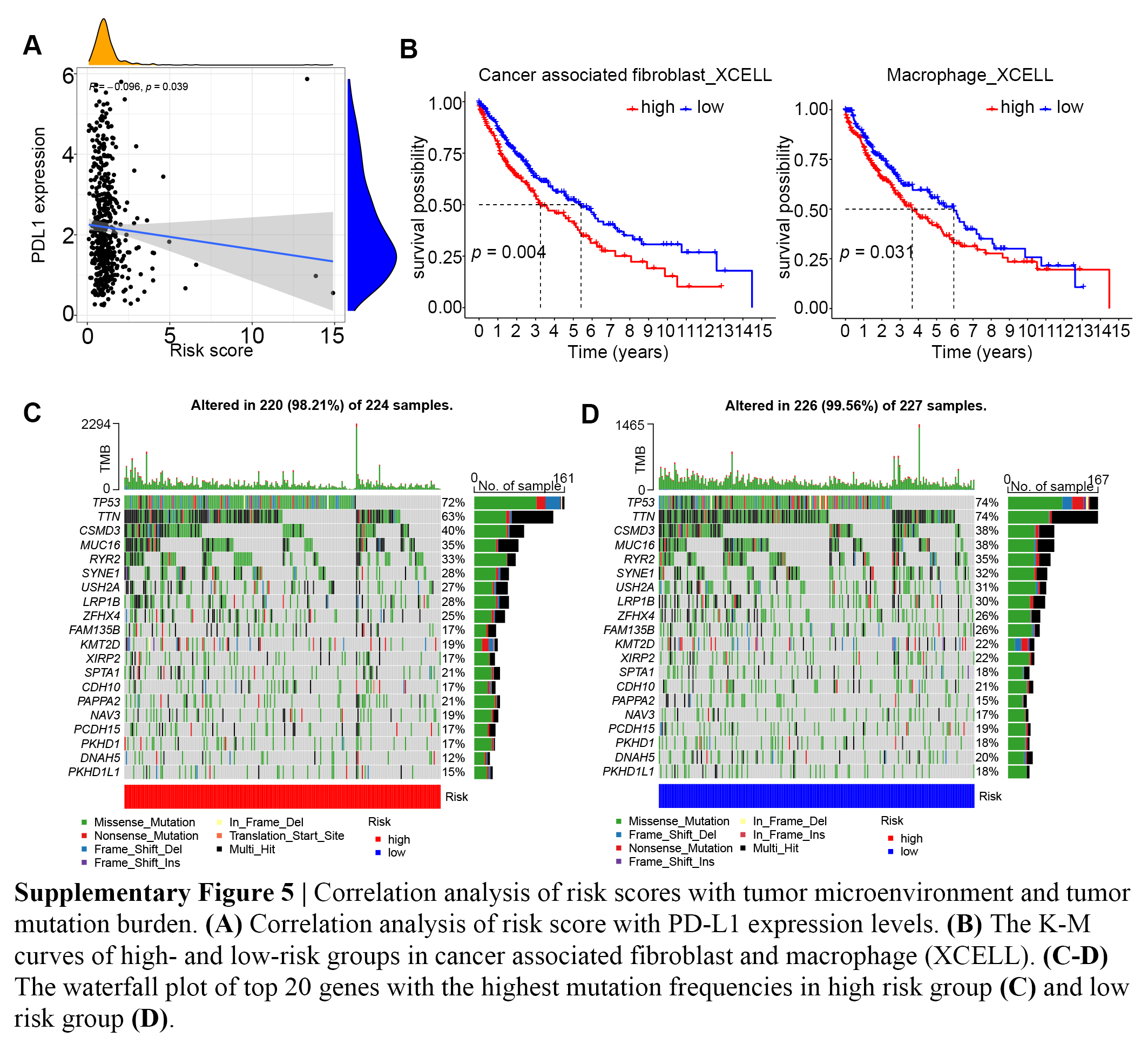

Supplement: Supplementary file 5 [file Image5.TIF]
